# Intelligent Manufacturing Systems in COVID-19 Pandemic and Beyond: Framework and Impact Assessment

**DOI:** 10.1186/s10033-020-00476-w

**Published:** 2020-08-28

**Authors:** Xingyu Li, Baicun Wang, Chao Liu, Theodor Freiheit, Bogdan I. Epureanu

**Affiliations:** 1 Department of Mechanical Engineering, University of Michigan, Ann Arbor, MI 48109 USA; 2 School of Engineering, Cardiff University, Cardiff, CF24 3AA UK

**Keywords:** Intelligent manufacturing system, COVID-19 pandemic, Industrial network, Supply chain disruption, Optimization

## Abstract

Pandemics like COVID-19 have created a spreading and ever-higher healthy threat to the humans in the manufacturing system which incurs severe disruptions and complex issues to industrial networks. The intelligent manufacturing (IM) systems are promising to create a safe working environment by using the automated manufacturing assets which are monitored by the networked sensors and controlled by the intelligent decision-making algorithms. The relief of the production disruption by IM technologies facilitates the reconnection of the good and service flows in the network, which mitigates the severity of industrial chain disruption. In this study, we create a novel intelligent manufacturing framework for the production recovery under the pandemic and build an assessment model to evaluate the impacts of the IM technologies on industrial networks. Considering the constraints of the IM resources, we formulate an optimization model to schedule the allocation of IM resources according to the mutual market demands and the severity of the pandemic.

## Introduction

Pandemics like COVID-19 have created a spreading and ever-higher threat to human health. High mortality and morbidity pose complex issues to industrial networks and manufacturing, including supply disruptions and demand-side shocks [1]. Supply disruptions are attributed to production disruptions of direct suppliers and global transportation due to health risks and quarantine policies. Outbreaks have quickly affected production and material supply of 938 of the Fortune 1000 companies due to the disrupted production of tier 1 or tier 2 suppliers in China [2]. A recent Chinese industrial survey reports that 82% of enterprises suffered a profit loss, and 62% have reduced labor to control operational costs [3]. Increasing panic among consumers and firms has distorted demand patterns and created market anomalies that has affected the ability to ramp-up production in some industries, i.e., in the medical equipment industry, and reduced operations in others, i.e., the aircraft industry. These emerging challenges stemming from COVID-19 require industrial networks to be robust to production disruptions and market environment changes, and the manufacturing system to be agile so that production capacity can be leveraged to control risks and to support the needs of prevention.

Intelligent manufacturing (IM) is a promising solution to improve production efficiency in light of the demands of this highly dynamic epidemic situation. IM is built on enabling complex and real-time decision-making within automated manufacturing assets, utilizing data from networked machines and sensors [4, 5]. Recent breakthroughs in information and communication technologies (ICTs), including the industrial internet of things (IIoT), digital twins, big data analytics, cloud computing, and artificial intelligence (AI), make the vision of IM practical. IM technologies also support the human in managing increasingly complex operations through predictive tools [6], automated design [7], AI-predictive maintenance [8], and adaptive planning [9]. These tools, collectively integrated into human-cyber-physical systems (HCPS) [10], have the promise to reduce health risks created by COVID-19. In this article, we propose a novel IM system framework to address the challenges and potential for existing IM technologies to fight pandemic and similar disruptions. This framework represents a production recovery paradigm for the ‘human-work-safely’. Because of the interconnected nature of an industrial network, relief of a production disruption in targeted network nodes holds promise for mitigating the overall impact on the entire supply chain and its ability to fulfill market demand during the ongoing COVID-19 or future pandemics. We further assess the impact of IM technologies on the industrial network for its ability to support production recovery. We propose an optimization model that helps determine the best IM implementation strategy that considers an epidemic’s severity, demand patterns, and the industrial network structure.

## IM Framework During Pandemic Outbreaks

**Figure 1:**
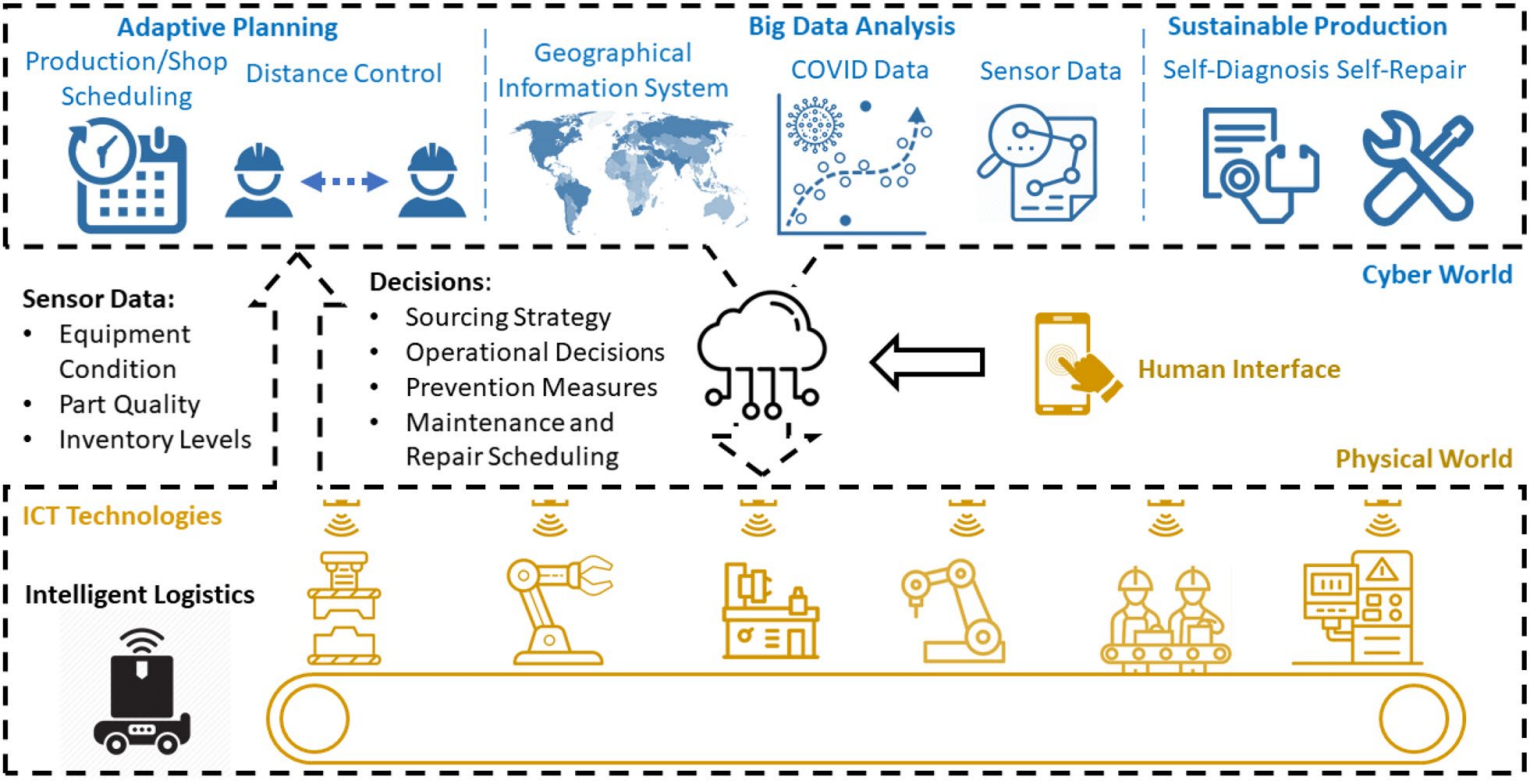
A framework of intelligent manufacturing systems in pandemic outbreaks

The proposed IM system combines the cyber world (computers, artificial intelligence, and networks) and the physical world (mechanical devices, equipment, sensors), and limited human workers in a closed-loop system. Figure 1 provides a schematic of an IM framework under pandemic outbreaks with connections to well-established IM techniques. The physical world is structured by automated assets, i.e., robots in the manufacturing line, autonomous vehicles with intelligent logistical control, and human operators. All assets are connected by ICT technologies with information on their status collected by sensors connected to the cloud. The cyber world involves a large number of multi-disciplinary methodologies to schedule operational decisions and epidemic prevention measures using data on equipment condition, part quality, and inventory levels, with an interface to the human decision-maker through a smart device. The information fusion and analysis are important for determining system performance and operational strategy. The critical applications of IM technologies to minimize production disruptions and safety risks in an epidemic are adaptive planning, big data analysis, and sustainable production.

### Adaptive Planning

Optimization algorithms and AI can realize functions in the cyber world such as supply and demand forecasting, design of manufacturing layout, capacity planning and configuration, and intelligent scheduling optimization. Epidemiological models can be integrated with these capabilities to help firms proactively shift their human resources, inventory levels, and sourcing strategies in factories, distribution centers, and consumer markets worldwide. The IIoT facilitates collaboration between manufacturing entities by sharing inventory and production plans in real-time, thus balancing capacity and demand [11]. Moreover, given precedence constraints in operations, system layouts and autonomous manufacturing assets can be adaptively adjusted to create safe spacing that separates human operators and minimizes health risks and the spread of disease. With sufficient computational power, production planning can be updated in real-time to adapt to the epidemic’s changing circumstances, thus an optimal policy can be adopted that balances production and health risks with profit objectives based on the latest status of the pandemic [12].

### Big Data Analysis

Big data analysis techniques are designed to explore and systematically extract information from large and diverse datasets with structured, semi-structured, or unstructured formats. In manufacturing, system performance is described from data that includes time-series signals, categorical variables, images, sounds, continuous sensor signals, and text, representing elements such as technical product characteristics to customer engagement and satisfaction surveys. External epidemic data together with Geographical Information Systems (GIS) can also be analyzed to predict the severity of the pandemic [13] and thus estimate the availability of components and raw materials from suppliers. Moreover, correlation analysis can reveal the impact of the pandemic severity on changes in market demand and production disruptions. With an estimate of the pandemic’s affect, a manufacturer can identify bottleneck suppliers during the recovery, trade-off product production volumes based on demand, and make decisions that improve cash flow and human resource utilization, and avoid material shortages.

### Sustainable Production

IIoT techniques and smart devices make remote equipment condition monitoring and maintenance scheduling practical. With AI and deep learning algorithms, advanced self-repair algorithms are able to diagnose product quality based on part images [14], identify the source of defects based on scheduled operations [8], and improve part quality through equipment and system reconfigurations [15]. An ability to self-repair minimizes the need for human effort in repeated inspection and testing, thus reducing health risks. Moreover, the travel needs of domain experts are considerably reduced, complying with pandemic travel bans and mitigating their exposure to risky environments.

## Impact Assessment

**Figure 2:**
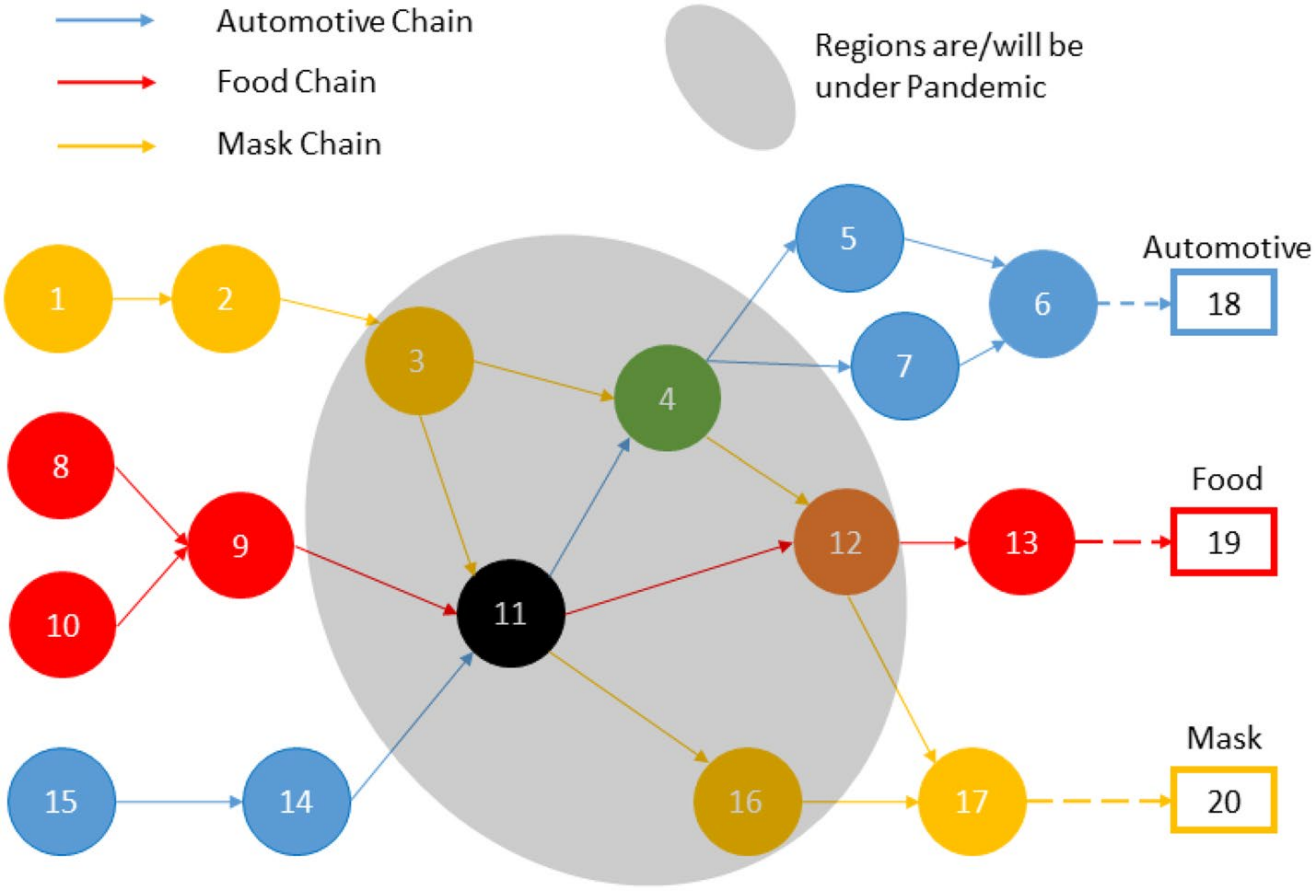
Hypothetical IIoT-based industrial network under a pandemic

The implementation of IM technologies benefits both the recovery of production in the manufacturing sector and facilitates the reconnection of the flow of goods and services to the industrial network, thus providing a profound influence on relieving disruptions [16]. Considering the urgency and limited IM resources available to implement IM during the current or future pandemics, determining an optimal priority for factory upgrades enhances the effectiveness for IM technologies to minimize damage to the industrial network and public health. Upgrade priority depends on the network structure, a forecast of the epidemic’s severity, and consumer demand patterns. The following assessment model shows the impact of IM technologies to disruptions. In this example, three industrial supply chains that deliver automotive, food, and health care personal protective equipment (masks) are considered. We aggregate the collective impact of IM techniques in their capacity to facilitate recovery from an outbreak. Figure 2 shows a hypothetical IIoT-based industrial network with circles representing factories, arrows representing material flows, and the pandemic region represented in grey.

The objective is to maximize production capacity of the end product by prioritizing which factories should be upgraded with an IM system based on changes in market demand, public health needs, and the pandemic severity in different regions. The industrial supply chain network is represented as graph $N = \left( {V,E} \right)$, with a set of source nodes $S = \left\{ {s_{1} , \ldots , s_{n} } \right\}$ being raw material providers, and a set of sink nodes $T = \left\{ {t_{1} , \ldots ,t_{m} } \right\}$ being end products to markets. The flow $f\left( {u,v} \right)$ of an edge $\left( {u,v} \right) \in E$ is the material flow between the factories. The base capacity $c\left( u \right)$ represents the maximum amount of production capacity that factory $u \in U$ can achieve in normal operation. When a factory $u$ is impacted by an epidemic, its capacity is calculated as $c\left( u \right) = \left( {1 - \alpha_{u} + i_{u} \beta_{u} } \right)c\left( u \right)$, where $i_{u}$ is a binary variable which is 1 when IM is applied and 0 otherwise, $\alpha_{u}$ is the capacity loss due to the epidemic, and $\beta_{u}$ is the capacity improvement and/or recovered due to the resilience that IM technology provides. To maximize the production capacity of all industrial supply chains, a linear optimization model is formulated as follows:

1 $$\mathop {\hbox{max} }\limits_{{f\left( {u,v} \right),i_{k} }} \mathop \sum \limits_{k = 1}^{k = m} w_{k} \mathop \sum \limits_{{u:\left( {u,t_{k} } \right) \in E}} f\left( {u,t_{k} } \right),$$

s.t.,

2 $$\mathop \sum \limits_{{v:\left( {u,v} \right) \in E}} f\left( {u,v} \right) = \mathop \sum \limits_{{v:\left( {v,w} \right) \in E}} f\left( {v,w} \right), \forall v \in \frac{V}{{\left\{ {S,T} \right\}}},$$

3 $$f\left( {u,v} \right) \le c\left( u \right), \forall u:\left( {u,v} \right) \in E,$$

4 $$\mathop \sum \limits_{u \in V} i_{u} \le b,$$

where $w_{k}$ represents the demand of end product $t_{k}$. Constraint (2) ensures the balance of the capacity flows between the adjacent manufacturers, which can be relaxed with consideration of inventory levels, constraint (3) ensures the flows cannot exceed the capacity of the factory, and constraint (4) is an upper bound on available IM resources, i.e., a limit, $b$, to the total number of factory upgrades possible. Note that constraint (2) represents a significant limitation to the simple example studied in this article. This constraint can be relaxed by using inventory levels and other methods already developed for a variety of manufacturing systems. In this example, we will set the network parameters as $\alpha = 0.2, \;\beta = 0.6, \;b = 5, \;c\left( u \right) = 10, \forall u$. The results for different scenarios, solved using the Simplex algorithm in the CPLEX package, are shown in Table 1.

**Table 1:**
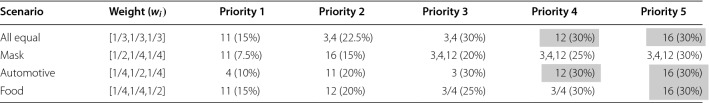
Performance improvement with changing demands under a pandemic

In Table 1, the columns indicate the priority of upgrade for the identified factory index, while the percent in the brackets shows the accumulated production recovery. For example, factory 12 is ranked fourth for IM technology upgrade in the automotive demand scenario, and the capacity recovery will be improved by 30% when factory 3 and its predecessors, i.e., nodes 4 and 11, are upgraded. The amount of capacity recovery increases with more IM technology implementations. Capacity also saturates such that more upgrades will not lead to a further improvement, i.e., once factory 3 is upgraded, no additional capacity is recovered in the automotive demand scenario, denoted by the gray shading. Note that factory 11 is ranked high in all demand scenarios due to its close connection to all of the industrial supply chains. Factory 4 shows higher priority in the automotive demand scenario, as factory 11 is not as vital an automotive supplier. The results show the responsiveness of the model to the changes in the demands pattern $w_{i}$, pandemic severity $\alpha$, and supply chain network $N$.

## Conclusions and Future Work

In this study, we explore the application of intelligent manufacturing (IM) as a proactive solution to mitigate production disruptions caused by a pandemic. A decision-making model is proposed for determining the optimal deployment of IM resources that strengthens an existing industrial network. Several promising research directions are recommended to address the depth of the IM development problem. First, advanced methods to connect factories through IIoT are needed to share inventory information. Second, a pandemic-driven job scheduling model with the objective of minimizing production risk due to the supply chain disruption and plant closures should be developed. This model, along with the IIoT connecting the factories, can provide guidance when reacting to a pandemic, e.g., switch to ventilator production or suspend certain product lines. To implement an industrial network model, big data analysis should be used to estimate the values of the pandemic impact factor α, which may vary in different industries or regions. The IM capability impact factor β, should also be determined, distinguishing the most critical IM capabilities when fighting a pandemic. Finally, worker health risks can be reduced by optimizing the factory layout and operations to maximize social distancing by mixing automated manufacturing assets and human operators. Further, implementing IM that minimizes capacity losses on products critical to fighting a pandemic will reduce societal health risks. This work provides a vision for the potential of IM and its implementation during pandemic outbreaks or similar future global or regional disruptions.
